# Case Report: An uncommon germline variant of familial GISTs: broadening the landscape of inherited GIST syndromes

**DOI:** 10.3389/fonc.2026.1662921

**Published:** 2026-02-12

**Authors:** Pallavi Roshini Ganesan, Max Vogel, Daniel Knight, Danielle Rosenzweig, Ross Michels

**Affiliations:** Prisma Health Department of Internal Medicine, Greenville Memorial Hospital, Greenville, SC, United States

**Keywords:** familial GIST, genetic testing, GI cancer, GIST, KIT activating mutations

## Abstract

Familial Gastrointestinal Stromal tumors (GIST) are rare neoplasms but GIST tumors are some of the most common soft tissue sarcoma subtypes. The incidence is between 10 to 15 cases per million worldwide and approximately 500 cases in the USA [1]. GIST is often diagnosed based on clinical presentation, the tumor’s anatomic location and immunohistochemistry (IHC) pattern [1]. Patients with familial GIST develop GIST tumors that are numerous, smaller in size, and occur in the background of intestinal cells of Cajal hyperplasia [2]. The first family with features of inherited GIST was reported in the 1990s which was the first time Exon 11 KIT variant was reported [3]. We present two cases of family members affected with multifocal GIST tumors who underwent germline genetic analysis and were found to have germline pathogenic variants in Exon 11 of the KIT gene [c.1735_1737del (p.Asp579del)]. Both patients were treated with Imatinib with good response.

## Introduction

Familial gastrointestinal stromal tumors (GISTs) are rare neoplasms, but GISTs are some of the most common soft tissue sarcoma subtypes. The incidence is between 10 and 15 cases per 1 million worldwide and approximately 500 cases in the USA (1). GIST is often diagnosed based on clinical presentation, the tumor’s anatomic location, and immunohistochemistry (IHC) pattern (1). Patients with familial GIST develop GISTs that are numerous and smaller in size and occur in the background of intestinal cells of Cajal hyperplasia (2). The first family with features of inherited GIST was reported in the 1990s, which was the first time that the exon 11 *KIT* variant was reported (3). We present two cases of family members affected with multifocal GISTs who underwent germline genetic analysis and were found to have germline pathogenic variants in exon 11 of the *KIT* gene [c.1735_1737del (p.Asp579del)]. Both patients were treated with imatinib with a good response.

## Case 1

**Figure 1:**
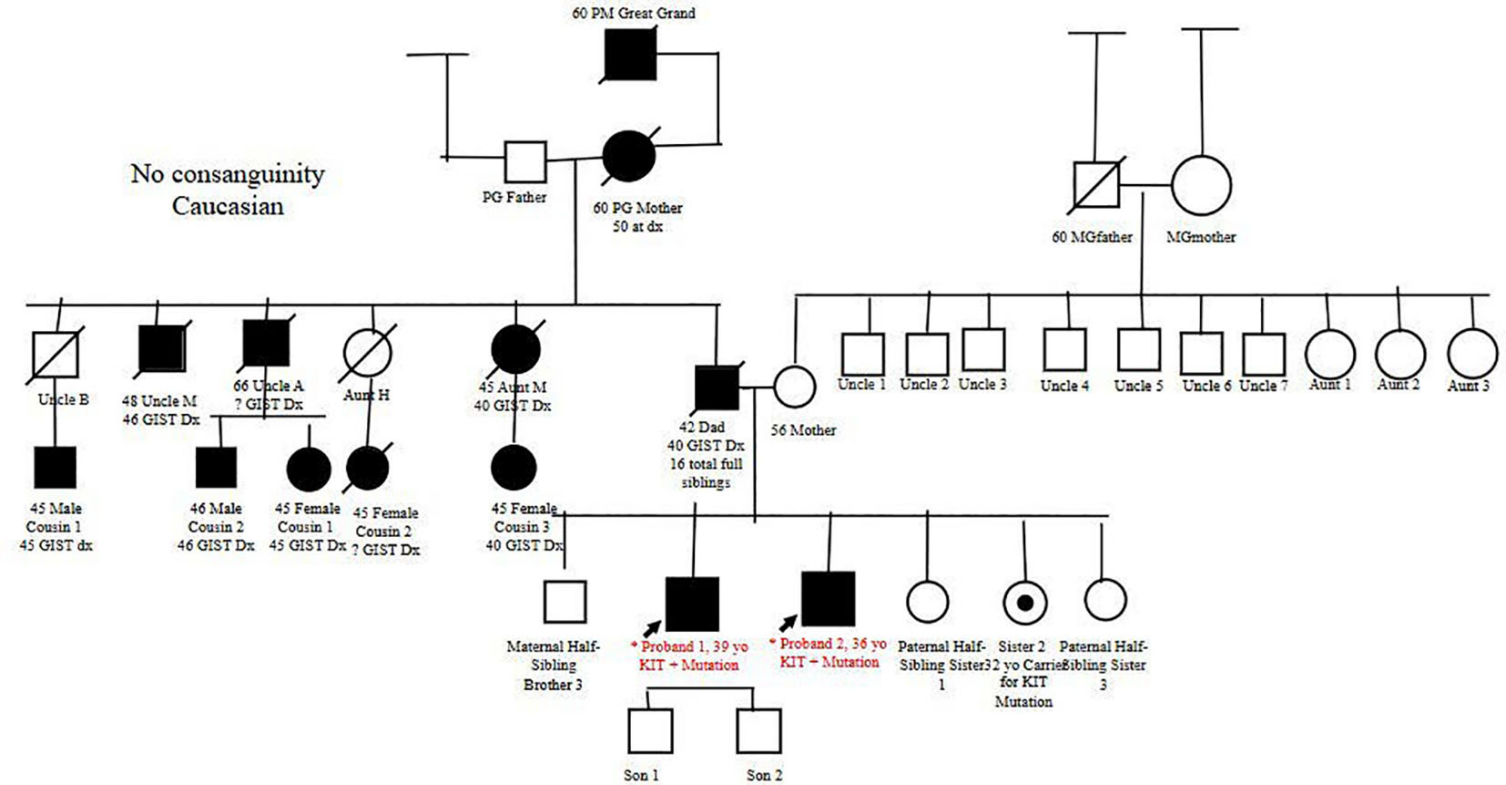
The pedigree of the family with confirmed relatives with GIST diagnosis of Probands 1 and 2 based on family history obtained from Proband 1. Family members were found to be positive for c.1735_1737del (p. Asp579del) pathogenic variant KIT (sequence identifier NM_000222.2). GIST, gastrointestinal stromal tumor.

Our first patient was a 39-year-old Caucasian man with a notable family history significant for GIST diagnosed in multiple family members, including his father, paternal grandmother, great-grandfather, great-uncle, two uncles, an aunt, and five first cousins (**Figure 1**).

**Figure 2:**
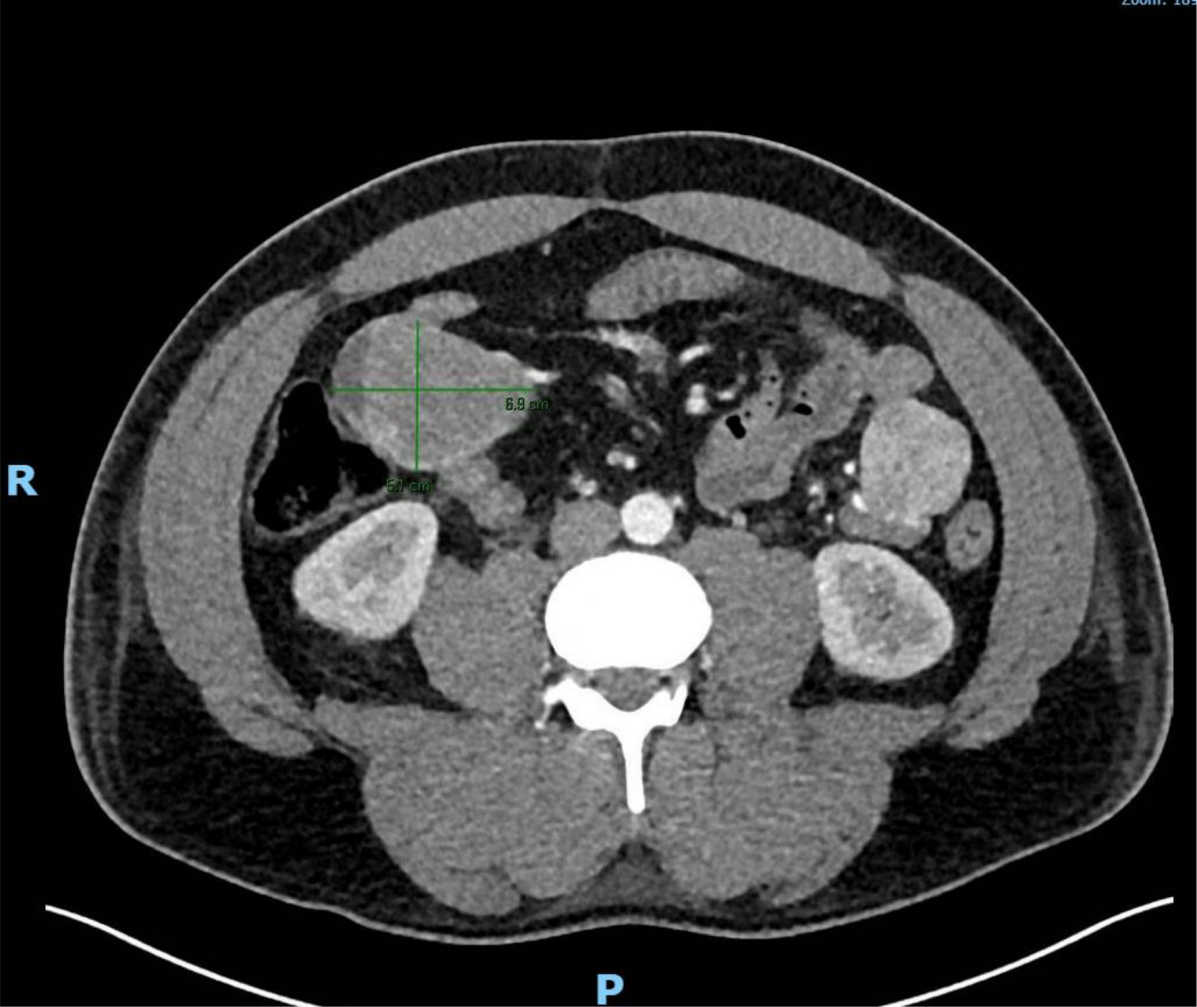
Largest hypervascular lesion sized 69 × 51 mm noted on CT angiogram of abdominal aorta (marked on image) in duodenum. Patient was noted to have 11 other hypervascular lesions in his duodenum.

In June 2023, our patient presented with black, tarry stool and shortness of breath with minimal exertion for 4 days. After a positive breath test, he was diagnosed with *Helicobacter pylori* infection and was prescribed triple therapy. During his workup, he was found to have hemoglobin of 3.5 g/dL. He received transfusion support and underwent emergent esophagogastroduodenoscopy (EGD). EGD revealed a submucosal mass in the third portion of the duodenum in addition to three arteriovenous malformations that were subsequently cauterized. The biopsy was initially benign due to deeper stromal mucosal tissue being unable to be obtained in the original EGD. Therefore, to further evaluate the bleeding source, the patient underwent a nuclear medicine GI bleed scan and abdominal computed tomography (CT) angiogram. The scans found 12 hypervascular lesions in the duodenum. The largest hypervascular lesion measured 69 × 51 mm in the right midabdomen (**Figure 2**).

After the patient was deemed a non-surgical candidate, he underwent interventional radiology (IR)-guided biopsy of duodenal lesions to obtain submucosal tissue. Pathology revealed a pathologic pT3M0N0 Grade 1 GIST tumor per the recent American Joint Committee on Cancer (AJCC) 8th edition criteria. The tumor was positive for somatic *DOG1* and *c-KIT* pathogenic variants. Staging CT and positron emission tomography (PET) were negative for distant disease. Given the extensive family history of GIST, the patient was referred for genetic counseling. Germline testing with Invitae Multi-Cancer + RNA 70 gene panel was performed, and the patient was determined to be heterozygous for the c.1735_1737del (p.Asp579del) pathogenic variant *KIT* (sequence identifier NM_000222.2). Consistent with prior reports of germline *KIT* pathogenic variants, the expression of GIST in the extended family displayed autosomal dominant inheritance with high penetrance. However, no extended family members had undergone germline genetic testing to the knowledge of the proband.

Based on prior published National Comprehensive Cancer Network (NCCN) guidelines, for patients with unresectable primary disease, initiation of a tyrosine kinase inhibitor such as imatinib is recommended. The patient started on imatinib 400 mg/day in March 2024, which was well-tolerated. However, in May 2024, the clinical course was complicated by several days’ history of dark black stools, which were found to have hemoglobin of 7.2, and the patient was found to have active bleeding from the tumor during which imatinib was pause.

CT indicated an occult GI bleed notable for jejunal hemorrhage, which required resection of a portion of the proximal to mid-jejunum, which contained approximately 17 sites of disease, ranging from 0.2 to 6.5 cm in the greatest dimension. The patient underwent an exploratory laparotomy with proximal to mid-jejunal resection in May 2024 and notably recovered well. He resumed imatinib in June 2024.

**Figure 3:**
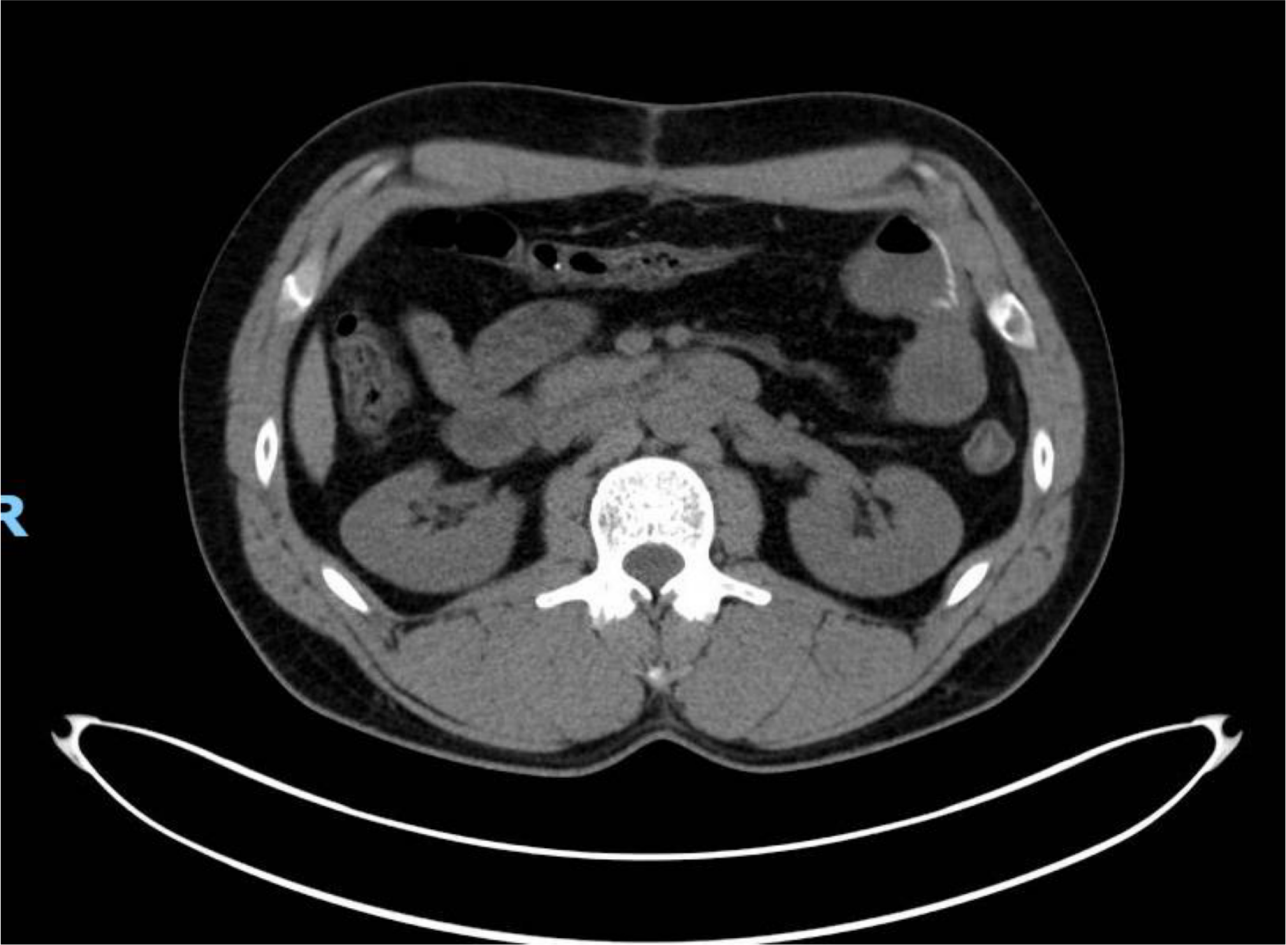
Positron emission tomography (PET) scan obtained after proximal to mid-jejunal resection and reinitiation of imatinib in July 2024. PET scan was notable for complete resolution of previous FDG-avid intra-abdominal masses seen in the CTA of abdomen in **Figure 2**.

Repeat PET/CT in July 2024 demonstrated a significant response to imatinib, with complete resolution of all previously noted 18F-Fluorodeoxyglucose (FDG)-avid intra-abdominal masses (**Figure 3**). The patient continued on imatinib with laboratory evaluation, imaging, and provider visits every 3 months. The patient’s two children underwent genetic testing and were negative for the KIT variant.

## Case 2

**Figure 4:**
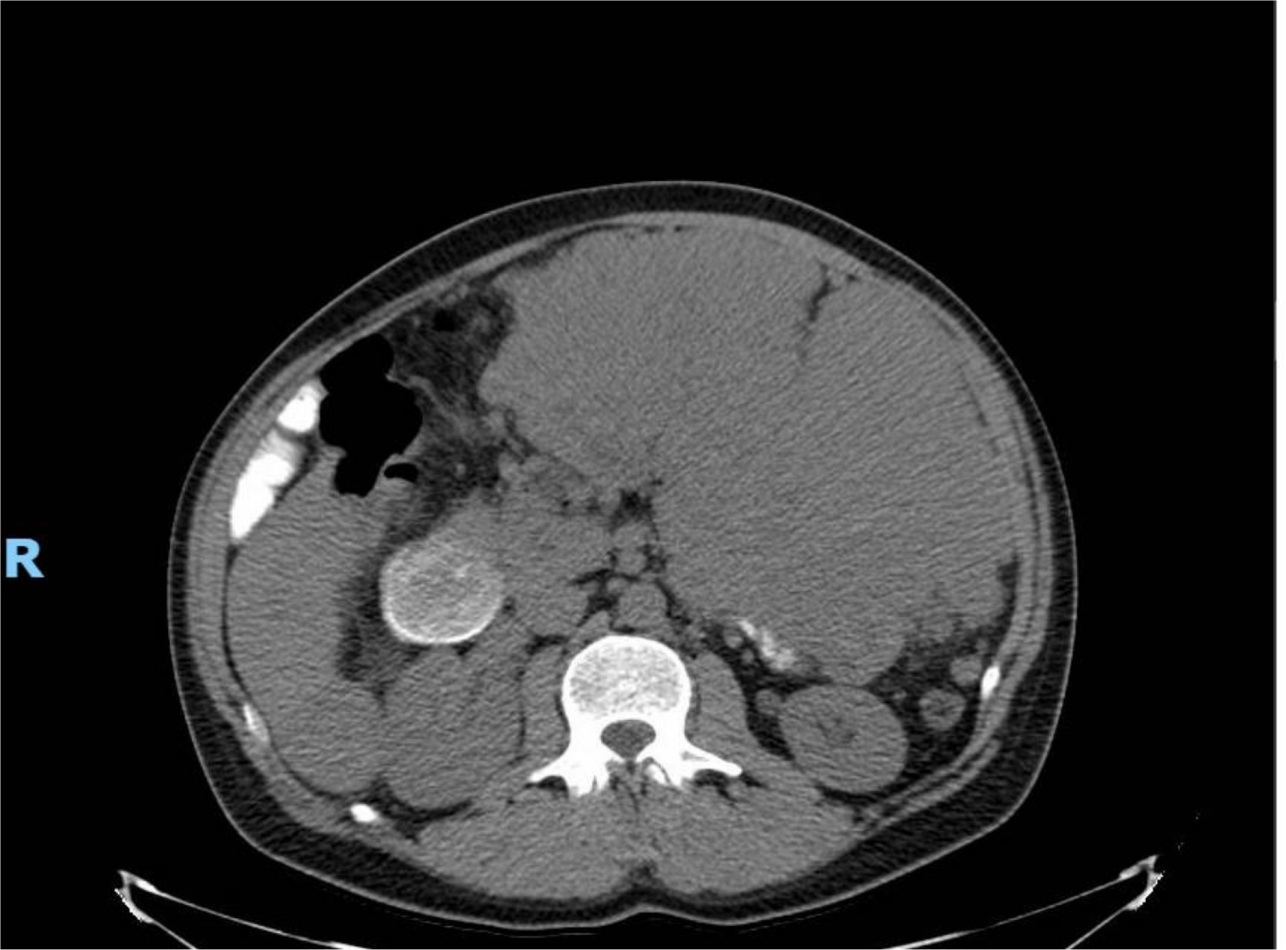
Initial CT of abdomen/pelvis with large mass, with largest component measuring 23 × 15 × 28 cm in left abdomen, with evidence of metastatic disease in the liver. Imaging notable for duodenal dilation and partial obstruction of small bowel.

Our second patient was the full brother of our first patient. This patient was a 36-year-old Caucasian man who originally presented to urgent care with 1 month of fatigue, anorexia, bloating, abdominal pain, and subcutaneous lumps of the abdomen. A CT of the abdomen and pelvis was obtained as part of his workup, which demonstrated a large mass filling the left abdomen with lesions in the liver consistent with metastatic disease. Imaging also demonstrated duodenal dilation with partial obstruction of the small bowel (**Figure 4**).

The patient was examined by surgical oncology in April 2024 and opted for palliative debulking of the dominant mass for relief of obstructive symptoms prior to beginning medical therapy. Given the extent of the disease, the patient required distal pancreatectomy, splenectomy, immobilization of the splenic flexure, omentectomy, transverse colon resection, duodenal resection, and partial left nephrectomy. A 30-cm maximal dimension portion of the dominant tumor was also removed during the procedure. The left colon was anastomosed to the right colon using a side-to-side anastomosis. Post-operatively, the patient tolerated the procedure well. He required total parenteral nutrition after which his diet was slowly advanced to liquids and solids.

Pathology of the resected 30-cm mass revealed pathologic pT4N1m, grade 2 GIST tumor (per AJCC 8th edition guidelines) with somatic *KIT* and *DOG1* pathogenic variants in two of 18 resected lymph nodes involved with the tumor. The spleen and pancreas were uninvolved by tumor, but multiple peritoneal implants and masses in the transverse colon and duodenum were confirmed to be GIST per pathology.

**Figure 5:**
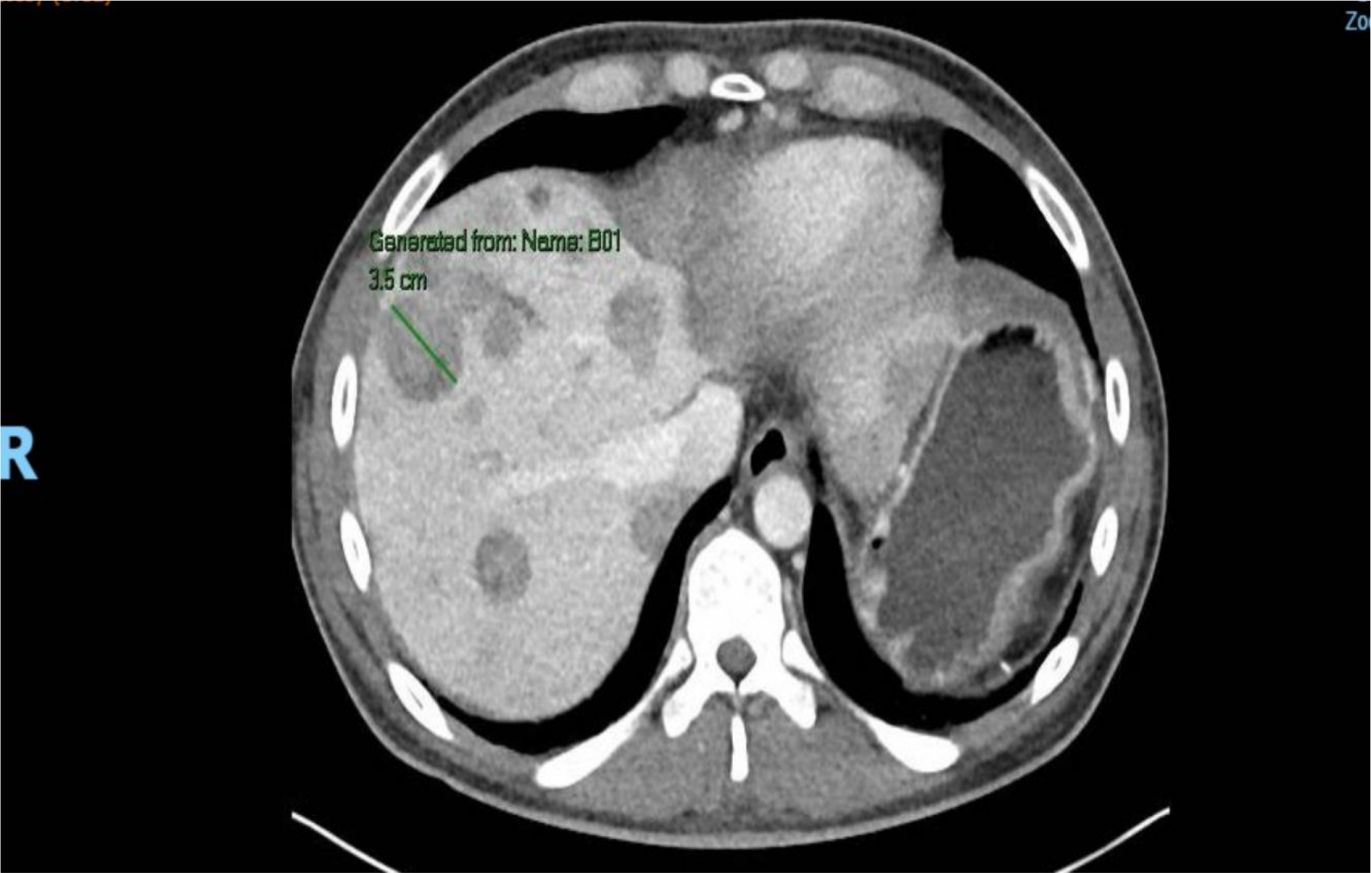
CT A/P post-debulking of mass and initiation of imatinib. Imaging showed notable improvement in hepatic metastasis in size and number.

Given the family history, the patient was referred for genetic counseling and confirmed to be heterozygous for the c.1735_1737del (p.Asp579del) germline pathogenic variant in *KIT*. Single-gene next-generation sequencing of *KIT* at the Invitae laboratory was performed due to the prior detection of this pathogenic variant in case 1. Per established NCCN guidelines, the patient was subsequently started on imatinib 400 mg/day in June 2024. At the time of diagnosis, he and his reproductive partner were considering treatment via reproductive endocrinology to achieve pregnancy, and at this time, we did not know if that was pursued. Imatinib was well tolerated, and the patient initially responded well to therapy. A CT scan in July 2024 showed a decrease in size of the majority of hepatic metastases. The largest hepatic lesion decreased in size to 6.6 × 4.2 cm, previously measuring 6.7 × 5.0 cm (**Figure 5**).

Unfortunately, in repeat screening imaging, CT of the chest/abdomen/pelvis noted stable hepatic metastasis but worsening portal hypertension with an increase in portal vein dilation, with significant ascites noted. Gastroenterology was consulted for which patient was started on Lasix and Aldactone.

During follow-up visits, our patient had been continued on imatinib with laboratory studies, imaging, and provider visits every 3 months, which had all been stable so far. Due to two family members having germline GISTs, the other four siblings underwent further genetic testing. Both probands had three half-siblings who did not undergo further testing. However, one of the full sisters (sister 2) was found to be a carrier for a germline variant of GIST. During the time of evaluation, this sister had no clinical manifestation notable for GIST. **Table 1** lists key characteristics of both probands.

**Table 1:** Summary of patient characteristics, treatment duration, and progression of both probands.

Proband	Demographics	Initial presentation	Treatment duration	Response
Proband 1	39-year-old Caucasian man	12 hypervascular lesions in the duodenum. The largest hypervascular lesion measured 69 × 51 mm in the right midabdomen	March 2024–current	After proximal to mid-jejunal resection in May 2024, noted to have complete resolution of all previously noted FDG-avid intra-abdominal masses.
Proband 2	36-year-old Caucasian man	23 × 15 × 28-cm mass in left abdomen with evidence of metastatic disease in the liver.	June 2024–current	After resection of mass, noted to have decrease in size of most hepatic metastases. The largest hepatic lesion decreased in size to 6.6 × 4.2 cm, previously measuring 6.7 × 5.0 cm.

## Discussion

GISTs are tumors that originally were thought to originate from the mesenchymal cells of the GI tract. However, it has recently been discovered that they arise from the cells of Cajal (4). They are cells positive for Kit and dependent on Kitligand (stem cell factor), located around the myenteric plexus and in the muscularis propria throughout the GI tract. These tumors stain positive for the CD117 antigen, also known as c-Kit proto-oncogene (5).

GISTs account for 1%–2% of all gastrointestinal neoplasms (4). GISTs can be found in the stomach (56%), small bowel (32%), colon and rectum (6%), esophagus (0.7%), or other locations (5.6%) (5). GISTs have three different histological findings, including spindle (70%), epithelioid (20%), and mixed types (10%) (4). The majority of GISTs stain positive for CD117 and DOG-1. Recent literature suggests that DOG-1 appears to be more sensitive and specific than CD117 (4).

Typically, patients present asymptomatically, and tumors are often incidental findings in abdominal CT scans. Patients with symptoms usually endorse non-specific symptoms such as nausea, vomiting, abdominal distention, early satiety, and rarely a palpable abdominal mass (4). The best modality to identify locations of GISTs is a CT enterography, but CT-guided biopsies provide a definite diagnosis (4).

Surgical resections remain the mainstay of treatment; however, the use of imatinib, a selective tyrosine kinase inhibitor (TKI), is commonly used as an adjuvant or neoadjuvant therapy (4). In a study by Wu et al., the majority of patients with GIST undergo resection with curative intent with the use of imatinib for metastatic disease (4). In patients with metastasis, patients are recommended to undergo serial CT scans every 3 months for 5 years (4).

Familial GISTs occur in patients with hereditary germline *KIT* or platelet-derived growth factor receptor alpha (*PDGFRA*) pathogenic variants (5). Mutually exclusive gain-of-function *KIT* or *PDGFRA* pathogenic variants occur in a majority of GISTs, representing in-frame deletions, point mutations, duplications, and insertions (5). Pathogenic variants in the *KIT* juxtamembrane domain (exon 11) are the most common in GISTs of all sites, whereas the rare *KIT* extracellular domain (exon 9) Ala^502^-Tyr^503^ duplication is specific for intestinal GIST. Exon 11 is the helical domain of *KIT*, which may represent the inhibitory region regulating *KIT* activation; therefore, disruption can impair auto-inhibition and lead to uncontrolled activation of cKIT protein. In-frame deletions of one to several codons have been noted in *KIT* exon 11 genes (5).

Affected individuals with germline *KIT* pathogenic variants demonstrate diffuse hyperplasia of their interstitial cells of Cajal. This is a unique feature, contrasting with the other germline pathogenic variants in other genes associated with GIST development, which provides supportive evidence of the defining role of c-KIT activation in the hereditary and sporadic setting (6).

In a literature review of 112 patients with familial GIST, it was found that the most common age of diagnosis was 41.5, with 77% of patients presenting with multifocal GIST at initial diagnosis (7). Two patients aged 36 and 39 were diagnosed with GIST, and both were found to have multifocal GIST.

Both of our patients presented with the c.1735_1737del (p.Asp579del) germline pathogenic variant in *KIT* that resulted in a deletion of one amino acid in the *KIT* gene, but preserved the reading frame, which is also known as 1756_1758delGAT and 1753del3. According to the PubMed database, there are a total of 49 families with hereditary GISTs due to germline KIT variants (7). However, there have only been 20 families reported in literature with variants that have arisen from specifically exon 11 of *KIT* (8–12). This specific variant [c.1735_1737del (p. Asp579del)] has only been reported in approximately six families, including the family reported in this paper.

The p.Asp579del variant is not within the region of high-frequency variants noted at positions 556 to 560 in exon 11 of *KIT* in patients with GIST, which is an additional unique factor to this presentation, as 11 of the 20 cases of familial GIST syndrome with variants in exon 11 of *KIT* were in this high-frequency region (13). In a study by Nakahara et al., they found that deletion at codon 579 of the c-kit gene resulted in a constitutive activation of the KIT gene, which was the first report of a gain-of-function variant in a region other than the Lys 550 to Val-560 region, which is relevant to our reported variant (14). This new variant in codon 579 was also discovered in a study by Tarn et al. (13).

Additionally, there have been some studies that suggest that patients with the variant of p.Asp579del, such as the patients in this report, may be at high risk for metastatic disease (15). One study theorized that patients noted to have a pathogenic variant in exon 11 were likely to have a stronger response to imatinib and decreased resistance to imatinib (16). In this study, nine of 11 family members who did not receive imatinib eventually passed from metastatic GIST complications, but the four family members who received imatinib achieved stable disease for 4+ years (16).

However, in a study by Antonescu et al. (17), they reported that patients with a primary KIT exon 11 variant were more likely to develop resistance via secondary variation in KIT exon 17. The median time of progression toward resistance was 24 months (17). Our probands did not undergo further testing for secondary variants beyond initial testing.

Currently, there is a paucity of standard guidelines for germline testing in GISTs. Based on the current 2025 NCCN guidelines, referral to a genetic counselor for germline testing is recommended in patients with a family history of GIST and/or melanoma, patients with multifocal GIST, and/or patients with NF1 or SDH-deficient GIST.

However, there have been studies in an attempt to further expand genetic testing. There have been prior studies that evaluated germline contributions in patients with syndromic features, given the assumption that GISTs resulting from germline alterations are frequently syndromic (18). In one study by Mandelker et al. (19), they found that most patients with germline pathogenic/likely pathogenic variants in the GIST-associated genes did not present with syndromic features, with 63% of patients with the germline variants also not having any family history of syndromic features. This suggests that a significant portion of KIT/PDGFRA wild-type GISTs that appear to be sporadic may have germline alterations that would previously not be identified. There are also no clear indications that exist for the role or extent of surgery in the setting of multifocal GISTs, as in our patients (8).

Additionally, there are very few clinical management guidelines for those who are found to be carriers of KIT pathogenic germline variants but have not developed any GISTs. In one study, a 53-year old patient was found to be a carrier of pathogenic GIST after her nephew was diagnosed. At the time of genetic testing, the patient had no clinical manifestations; however, she underwent a full-body MRI, where she was noted to have multifocal GISTs and underwent treatment with imatinib (18). Due to the paucity of guidelines, some studies, such as Bachet et al., recommend a follow-up of CT or MRI scans every 2–3 years in unaffected carriers or affected patients with small or asymptomatic GISTs (20). In many cases, patients with hereditary predispositions to GIST can have an indolent presentation and remain asymptomatic depending on the burden of disease (18). In our case, the sister of our two probands was noted to be a carrier of a variant of GIST; however, we were unable to obtain further data on specific genetic variants. She was recommended to visit a hematologist/oncologist for close monitoring in the setting of being a carrier.

There remain some limitations to this case report. We were only able to obtain a family pedigree based on Proband 1’s information, which leaves multiple family members out of the pedigree. Additionally, both probands were only tested for KIT variants and did not undergo testing for further variants, such as secondary variants.

In summary, we report a case of two brothers noted to have a rare genetic variant in exon 11 resulting in a deletion at position 579 and presented with multifocal GISTs. Based on the presented information, we hope to add to the growing body of research to help better understand the epidemiology, characteristics, and treatment of pathologic familial GISTs.

## Data Availability

The original contributions presented in the study are included in the article/supplementary material. Further inquiries can be directed to the corresponding author.
